# Human Wharton’s jelly-derived mesenchymal stromal cells reduce renal fibrosis through induction of native and foreign hepatocyte growth factor synthesis in injured tubular epithelial cells

**DOI:** 10.1186/scrt215

**Published:** 2013-06-04

**Authors:** Tao Du, Xiangyu Zou, Jun Cheng, Shuai Wu, Liang Zhong, Guanqun Ju, Jiang Zhu, Guohua Liu, Yingjian Zhu, Shujie Xia

**Affiliations:** 1 Department of Urology, Shanghai First People’s Hospital, School of Medicine, Shanghai Jiao Tong University, No 100, Haining Road, Shanghai, 200080, PR China; 2 Department of Urology, Nantong Tongzhou People’s Hospital, Nantong, Jiangsu Province, PR China; 3 Department of Urology, Qingdao Municipal Hospital, Qingdao, PR China

## Abstract

**Introduction:**

Based on some well-documented reports, we attempted to clarify the antifibrotic mechanisms of human Wharton’s-jelly-derived mesenchymal stromal cells (WJ-MSCs) from the perspective of induction of hepatocyte growth factor (HGF) expression in tubular epithelial cells (TECs).

**Methods:**

A rat model of acute kidney injury (AKI) was established through unilateral renal ischemia for 1 hour. Two days later, a single intravenous cell or vehicle injection, or contralateral nephrectomy, was performed. Rats were sacrificed at 1 day, 1 week, 4 weeks, or 6 weeks after the intervention. Renal fibrosis was evaluated by Masson trichrome staining and Sircol collagen assay. The upregulation of α-smooth muscle actin (α-SMA) versus E-cadherin expression was adopted as an indicator of tubular epithelial-mesenchymal transition (EMT). Gene and protein expression of HGF or transforming growth factor-beta1 (TGF-β1) was determined by real-time polymerase chain reaction (RT-PCR) and Western blot, respectively. HGF expression in TECs was detected with immunostaining. *In vitro*, rat TECs subjected to hypoxia injury were incubated with or without conditioned medium (CM) from WJ-MSCs for 1, 3, 24, or 48 hours. Rat or human HGF synthesis in TECs was assessed with immunostaining, RT-PCR, or ELISA.

**Results:**

Cell delivery or nephrectomy led to abrogation of renal scarring. At the incipient period of AKI, through induction of HGF expression, either of them remarkably promoted the upregulation of HGF versus TGF-β1 expression in damaged kidney. Rat TECs were not only the principal cells expressing HGF but also exhibited human HGF expression after cell infusion. During fibrogenesis, the downregulation of HGF versus TGF-β1 expression was greatly prevented by WJ-MSCs or kidney removal, thereby resulting in tubular EMT delay. *In vitro*, after 24 or 48 hours of incubation, CM not only robustly induced the upregulation of rat *HGF* gene expression in TECs but substantially amplified the release of rat HGF. Under the induction of CM, human HGF mRNA and protein were detected in rat TECs.

**Conclusions:**

WJ-MSCs contribute to tubular EMT delay and the alleviation of renal fibrosis. Induction of native and foreign HGF synthesis in damaged TECs at the initial stage of AKI leads to recovery of the disturbed balance of HGF/TGF-β1 during scar formation, being one of the vital mechanisms.

## Introduction

AKI can result in proliferation of fibroblasts and excessive deposition of extracellular matrix [1] and has been recognized as a major contributor to end-stage kidney disease [2]. The mechanisms implicated in AKI-induced kidney scarring remain controversial. Tubular EMT has been proposed to one of crucial cellular mechanisms [3-5]. One maneuver abrogating tubular EMT during injury will halt the progression of renal fibrosis.

TGF-β1, as a prototypic inducer of tubular EMT, can initiate and complete the entire course of EMT [6]. By contrast, HGF can mechanistically inhibit the EMT through blockade of Smad signaling, the downstream of TGF-β1 [7,8]. Hence, the delicate balance between HGF and TGF-β1 may have an influence on tubular EMT.

The role of MSCs in accelerating AKI recovery has been appreciated for a long time. It is generally thought that the administration of MSCs exerts antiinflammatory, proproliferative, antiapoptotic effects by paracrine/endocrine mechanisms [9-11]. Soluble factors and microvesicles (MVs) released by MSCs, are acknowledged as the vital mediators of these effects [12]. Because of the benefit of HGF in AKI [13], we have great interest in its role played in the sophisticated mechanisms of MSCs. In our previous study, WJ-MSCs, an alternative source of MSCs, mitigate AKI through delivery of exogenous HGF and induction of HGF gene expression in damaged kidney tissue [14]. Given that MVs can affect gene transcription of target cells through the transfer of genetic information (including transcription factor and mRNA) [10], we hypothesized that WJ-MSCs may be involved in the modulation of the HGF/TGF-β1 balance through induction of native and foreign HGF synthesis in injured renal TECs (target cells), thereby stopping tubular cell phenotype transition.

The aim of the present study was to test this hypothesis in a rat model of unilateral ischemia-reperfusion injury (IRI). We demonstrated that WJ-MSCs can restore the disturbed balance of HGF/TGF-β1 during fibrogenesis via induction of native and foreign HGF synthesis in host renal TECs at the initial stage of ischemic AKI, as a result of which tubular EMT delay and rescue of renal fibrosis occur. To the best of our knowledge, this is the first article to disclose the antifibrotic mechanisms of MSCs from the perspective of their impact on HGF expression in renal tubular cells.

## Methods

### Preparation of WJ-MSCs

Fresh human umbilical cords that are usually discarded after delivery were obtained with the written consent of the parents. This experiment was approved by the Research Ethics Committee at Shanghai Jiaotong University First People’s Hospital (Permit number: 2013KY001). WJ-MSCs were prepared and identified as described previously [14]. In brief, mesenchymal tissues were cut into 1-mm^2^ pieces and then stuck to the substrate of culture plates individually, followed by the addition of low-glucose Dulbecco Modified Eagle Medium (DMEM) containing 10% fetal bovine serum (FBS). About 12 days later, the colonies appeared and cultured on new plastic plates for further expansion. The cultured cells expressed the markers of MSCs (CD44, CD73, CD90, and CD105) with no expression of hematopoietic and endothelial markers, and had the potential to differentiate toward chondrocytes and osteoblasts. The isolated cells fulfilled minimal criteria for defining MSCs by the International Society of Cellular Therapy (ISCT) [15]. The cells at the third to sixth passage were used in *in vivo* and *in vitro* experiments.

### Animals

All works involving animals were in accordance with the animal use protocol enacted by the Institutional Animal Care and Use Committees of School of Medicine, Shanghai Jiaotong University. Adult male Sprague–Dawley (SD) rats weighing 180 g to 200 g were housed at a constant temperature and humidity, with a 12:12-hour light–dark cycle, and were allowed ree access to a standard diet and water. After the operation, rats were housed individually in a ventilated cage system.

### Animal model of unilateral renal ischemia-reperfusion injury

Under induction of isoflurane, IRI animals were subjected to left kidney ischemia for 60 minutes. Two days later, sham operation was performed followed by the intravenous infusion of 2 × 10^6^ WJ-MSCs in 0.5 ml serum-free medium (SFM) (vehicle), whereas control animals received 0.5-ml vehicle infusion instead of the cells. IRI animals undergoing intact kidney removal were regarded as the positive control. Sham-operated animals did not experience ischemic injury. The animals were randomized according to different therapeutic procedures: (a) sham-operated animals (*n* = 24); (b) unilateral IRI plus vehicle-injected animals (*n* = 24); (c) unilateral IRI plus cell-injected animals (*n* = 24); and (d) unilateral IRI plus nephrectomized animals (n = 24). The animals were killed at 1 day, 1 week, 4 weeks, and 6 weeks after intervention, respectively. Blood and tissue samples (including kidney, lung, and liver) were obtained at death and submitted to corresponding evaluation.

### Tissue collagen concentration

The total soluble collagen concentration within each renal tissue sample was determined with Sircol collagen assay kit (Biocolor, Carrickfergus, Northern Ireland, UK) according to the manufacturer’s protocol. Tissue samples were dissolved in 0.5 *M* acetic acid and pepsin at 4°C. Through centrifugation of tissue suspensions, the supernatants were collected. The concentration of total collagen was measured at 546 nm.

### Masson trichrome staining

The degree of interstitial fibrosis was scored semiquantitatively on a 0-to-3 scale (0, no lesion; 1, <33% of parenchyma affected by the lesion; 2, 33% to 67% of parenchyma affected by the lesion; 3, >67% of parenchyma affected by the lesion). The scores were assessed by a blinded observer in 100 random high-power fields (HPFs) (magnification ×400) of parenchyma for each rat (*n* = 3 rats, each group). Total score was obtained by the addition of all scores, with a maximum score of 300.

### Immunohistochemistry staining

The 5-μm-thick paraffin-embedded sections were labeled with rabbit antibody to human nuclear mitotic apparatus protein (NuMA) (dilution, 1:50; Abcam, Cambridge, UK), rabbit antibody to rat or human HGF (dilution, 1:250 or 1:500; Abcam), mouse antibody to rat α-SMA (dilution, 1:500; Abcam), or rabbit antibody to rat E-cadherin (dilution, 1:500; BD Biosciences, Franklin Lakes, NJ, USA) followed by HRP-conjugated secondary antibody by using 3,30 diaminobenzidine (DAB) reagents as substrate. Negative control was performed by omitting primary antibodies. Harris hematoxylin counterstaining was performed. All the sections were reviewed by a blinded doctor from a pathology discipline.

### Western blot

Protein extracts (30 μg per lane) were electrophoresed and then transferred to polyvinylidene fluoride membrane. Immunoblotting was performed by incubating each membrane with an anti-HGF (dilution, 1:1,000; Abcam), TGF-β1 (dilution, 1:1,000; Abcam), α-SMA (dilution, 1:1,000; Abcam), E-cadherin (dilution, 1:2,500; BD Biosciences), or β-actin antibody overnight at 4°C. After being washed in PBS, each membrane was incubated for 1 hour with a secondary antibody conjugated by peroxidase at room temperature. The band was developed by use of enhanced chemiluminescence (Amersham Pharmacia Biotech, Piscataway, NJ, USA). The density of each band was determined. The results were repeated twice to confirm the reproducibility.

### Real-time PCR

Total RNA was extracted with the TRIzol Reagent (Invitrogen, Carlsbad, CA, USA) according to the standard protocol. Five micrograms of RNA was reverse transcribed with the M-MLV reverse transcriptase kit and Oligo dT primers (Invitrogen) for 60 minutes at 42°C. Real-time PCR was performed with TaqMan gene expression assays (Applied Bio-Systems, Foster City, CA, USA) for detection of gene expression of HGF, rat HGF, rat TGF-β1, human HGF, or human or rat β-actin. Real-time PCR was carried out by using the following primers: *HGF*, 5′tgacatcactcccgagaact, 3′caatagcaccgttaccctt; rat *HGF*, 5′cctatttcccgttgtgaag, 3′gtcatcccacctaccaatca; rat *TGF-β1*, 5′gaaggacctgggttggaagt, 3′gagatgttggttgtgttgggc; rat *β-actin*, 5′cctctatgccaacacagt, 3′gacacacctaaccaccga; human *HGF*, 5′CTCTGGTTCCCCTTCAATAG, 3′GATAGCCCCATTTCTGGATGTC; human *β-actin*, 5′AAGGTGACAGCAGTCGGTT, 3′GGAGAGGGTTCAGGTGTGT.

The Ct (threshold cycle) for each gene was determined for each sample. The quantification of the target gene was normalized by β-actin. The values were expressed relative to a reference sample (samples from sham-operated rats or TECs without exposure to CM). The relative mRNA expression was calculated by 2^-ΔΔCT^. Triplicates of each sample were performed.

### Analyses of HGF synthesis in injured-rat TECs

For the preparation of CM, WJ-MSCs were cultured overnight in SFM (low-glucose DMEM) (Life Technologies, Carlsbad, California, USA). Cell supernatants were collected and subjected to centrifugation at 2,000 *g* for 20 min to remove cell debris.

TECs (Sciencell, San Diego, CA, USA) were maintained in epithelial cell medium (EpiCM) (Sciencell), containing 2% FBS, until the cells were 80% confluent. For assessment of HGF production in rat TECs, the cells were cultivated in a humidified atmosphere containing 5% O_2_ and 5% CO_2_ at 37°C for 1 hour (Incubator, Binder, Germany) and then incubated with or without CM under ambient oxygen concentration (21%). After 24 or 48 hours of incubation, cell number was estimated with a hemacytometer, whereas trypan blue exclusion was used to assess cell viability. The supernatants and cells were harvested. HGF level in the supernatants was measured with a rat HGF ELISA Kit (R&D Systems, Minneapolis, MN,USA), whereas total RNA extracted from TECs was submitted to real-time PCR for evaluation of rat HGF gene expression. The ELISA results were normalized by cell numbers in culture.

For ascertainment of the delivery of human HGF mRNA from CM into rat TECs, the hypoxia-injured TECs were exposed to CM for 1, 3, 24, or 48 hours. These cells were collected and submitted to real-time PCR for human HGF mRNA detection. TECs without exposure to CM and WJ-MSCs were used as negative and positive controls, respectively. Moreover, the cells were submitted to immunochemistry staining for human HGF protein expression in TECs (described later).

All samples were frozen at -80°C until analysis. All experiments were performed in triplicate.

### Immunochemistry staining for human HGF expression in rat TECs

TECs injured by hypoxia were incubated on chamber slides and exposed to CM or control medium for 24 hours or 48 hours. Subsequently, the slides were fixed in 4% paraformaldehyde and permeabilized with HEPES-Triton X100 buffer (Sigma, St. Louis, MO, USA). Rabbit anti-human HGF antibody (dilution, 1:100; Abcam) was used as the primary antibody. In the negative control, the primary antibody was omitted. Harris hematoxylin was added for nuclear counterstaining. WJ-MSCs and rat TECs without exposure to CM were used as positive and negative controls, respectively.

### Statistical analysis

Data are expressed as mean ± SD. Primary data collection used Excel, and statistical analyses were carried out by using Prism software (Graph Pad, San Diego, CA, USA). Analysis of variance (ANOVA) or Student *t* tests were used to assess differences between data, as appropriate. A *P* value of <0.05 was considered significant.

## Results

### No WJ-MSCs reside in ischemic kidney at any given time

Through immunohistochemistry staining for human NuMA, WJ-MSCs were exclusively detected in lung tissues at 1 day and 1 week after infusion, whereas no positive-staining cells were detectable in damaged or normal kidneys, or other tissues (liver or spleen) at any point (1 day; 1, 4, or 6 weeks) (data not shown), implying an endocrine mechanism in favor of the notion proposed by Bi *et al.*[16].

### Cell treatment mitigates renal fibrosis triggered by unilateral IRI

**Figure 1:**
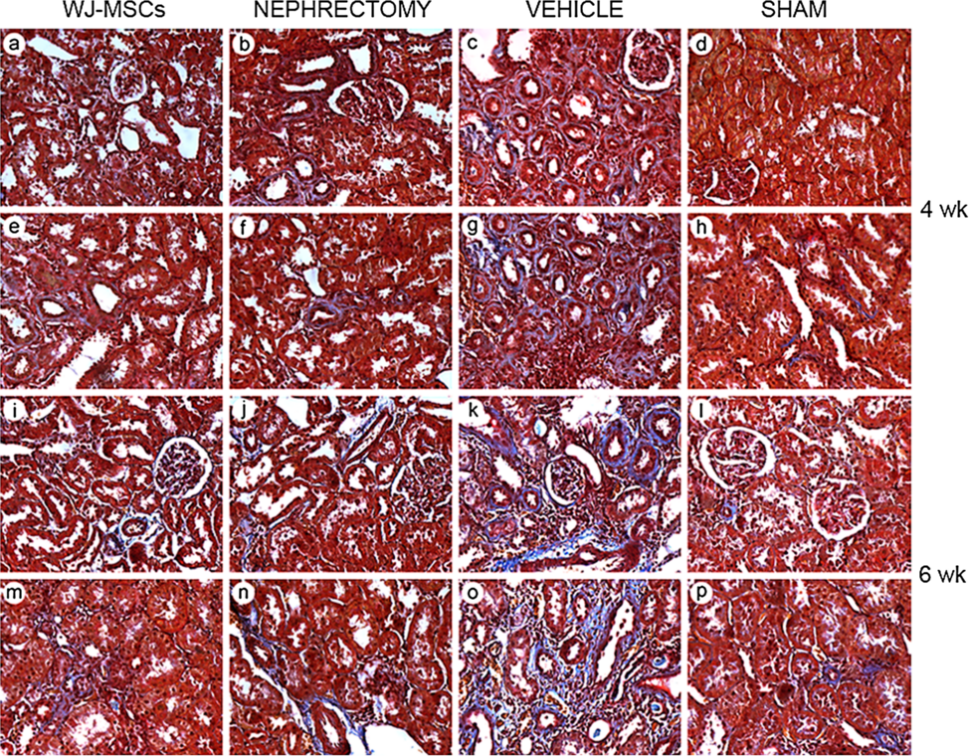
**Representative micrographs of Masson trichrome staining in tubular interstitial area.** Ischemic injury caused a progressive increase in positive-staining collagen deposition, whereas either nephrectomy or cell injection resulted in a remarkable reduction in collagen deposition. The original magnification is ×400.

**Figure 2:**
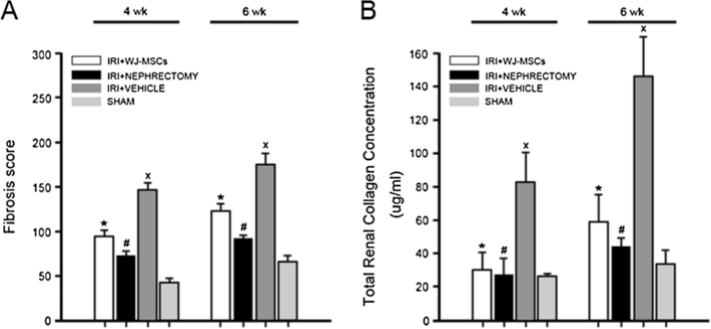
**Quantitative evaluation of collagen deposition in kidney tissues. (A)** Fibrosis score. The highest fibrosis score was achieved by vehicle-injected IRI rats, whereas cell-injected rats or nephrectomized rats had a significantly lower score. The fibrosis score was obtained by the addition of all scores for collagen staining from 100 random high-power fields (HPFs; magnification, ×400), with a maximum score of 300. All quantitative data were collected from three rats for each experimental condition. ∗*P* < 0.01, IRI+WJ-MSCs versus IRI+VEHICLE; #*P* <0.005, IRI+NEPHRECTOMY versus IRI+VEHICLE; x*P* < 0.001, IRI+VEHICLE versus SHAM; **(B)** Total renal collagen concentration. At either 4 weeks or 6 weeks, the highest collagen concentration was detected in kidney samples of IRI animals receiving vehicle injection, whereas cell injection or nephrectomy caused a marked decline in collagen concentration in damaged kidney tissues. All quantitative data were obtained from three animals for each experimental condition. ∗*P* < 0.02, IRI+WJ-MSCs versus IRI+VEHICLE; #*P* < 0.02, IRI+NEPHRECTOMY versus IRI+VEHICLE; x*P* < 0.05, IRI+VEHICLE versus SHAM.

At 4 or 6 weeks, either Masson trichrome staining or Sircol collagen assay revealed a marked increase in collagen deposition in ischemic kidney, whereas cell treatment or contralateral nephrectomy led to a remarkable decline in collagen deposition (Figures 1 and 2).

### The tubular EMT process triggered by unilateral IRI is delayed by cell delivery

**Figure 3:**
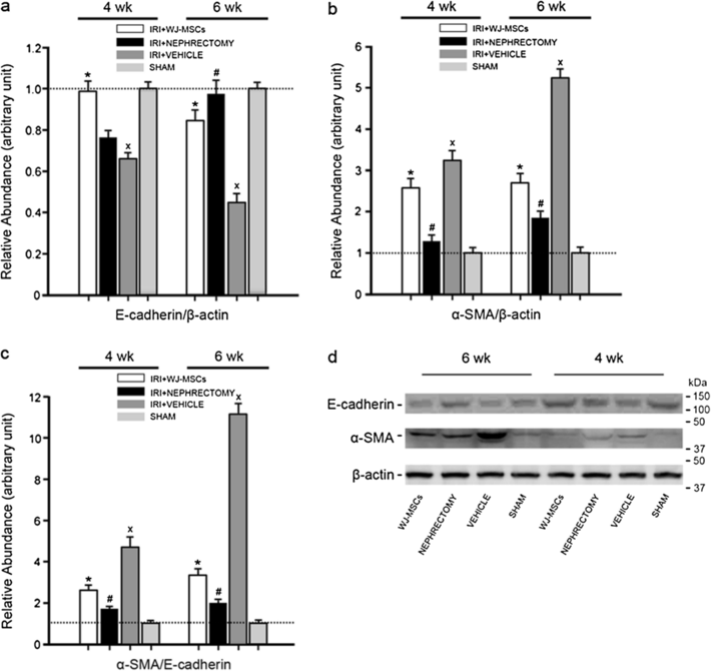
**Densitometric analysis and gel photograph of E-cadherin and α-smooth muscle actin (SMA) expression in kidney tissues.** Ischemic injury initiated the time-dependent upregulation of α-SMA/E-cadherin expression, indicative of the progression of EMT. This alteration was greatly prevented by cell treatment or intact kidney removal. The density of each band was determined. Values in the graph are expressed as densitometric ratios of E-cadherin/β-actin, α-SMA/β-actin, or α-SMA/E-cadherin as folds over control (sham-operated samples) (dotted line). Data are shown as mean ± SD of three kidney samples for each experimental condition. ∗*P* < 0.05, IRI+WJ-MSCs versus IRI+VEHICLE; #*P* < 0.01, IRI+NEPHRECTOMY versus IRI+VEHICLE; x*P* < 0.01, IRI+VEHICLE versus SHAM. **(a)** relative abundance of E-cadherin/β-actin; **(b)** relative abundance of α-SMA/β-actin; **(c)** relative abundance of α-SMA/E-cadherin; **(d)** gel photograph of E-cadherin, α-SMA, and β-actin protein expression.

**Figure 4:**
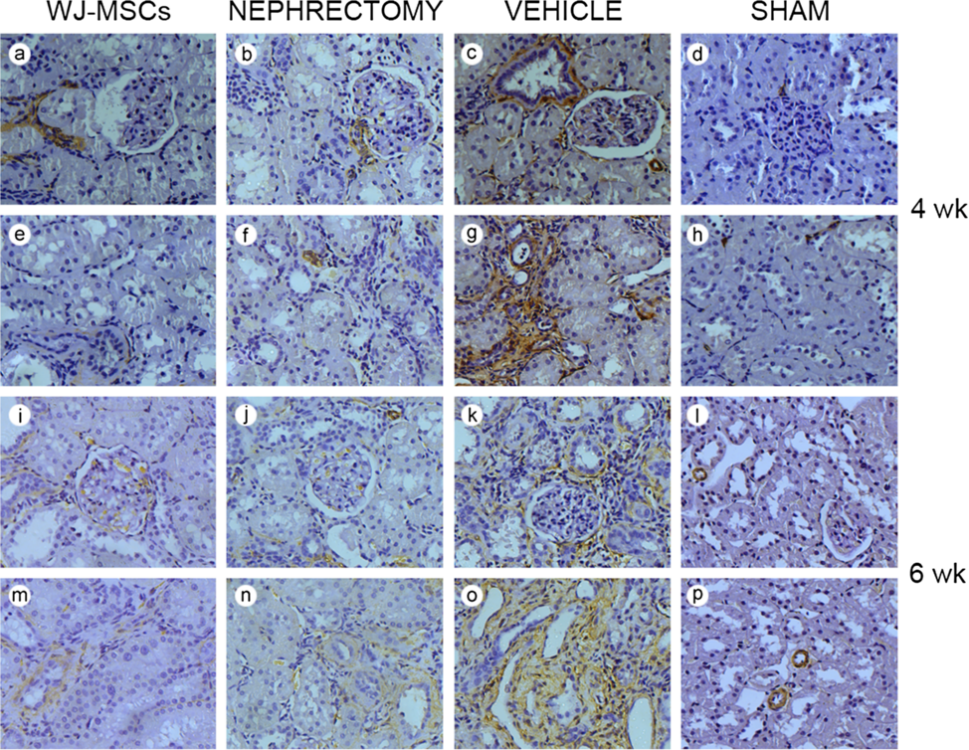
**Representative micrographs illustrating α-smooth-muscle actin (SMA) expression in kidney tissues.** In comparison with IRI rats receiving cell treatment or nephrectomy, vehicle-treated rats exhibited stronger positive staining for α-SMA in kidney tissue sections, especially at 6 weeks after intervention. In kidney tissue sections from sham-operated rats, α-SMA was expressed mostly in vessels. The original magnification is × 400. **(a)** through **(h)**, photographs showing α-SMA expression in kidney tissue sections at 4 weeks after intervention; **(i)** through **(p)**, photographs showing α-SMA expression in kidney tissue sections at 6 weeks after intervention.

**Figure 5:**
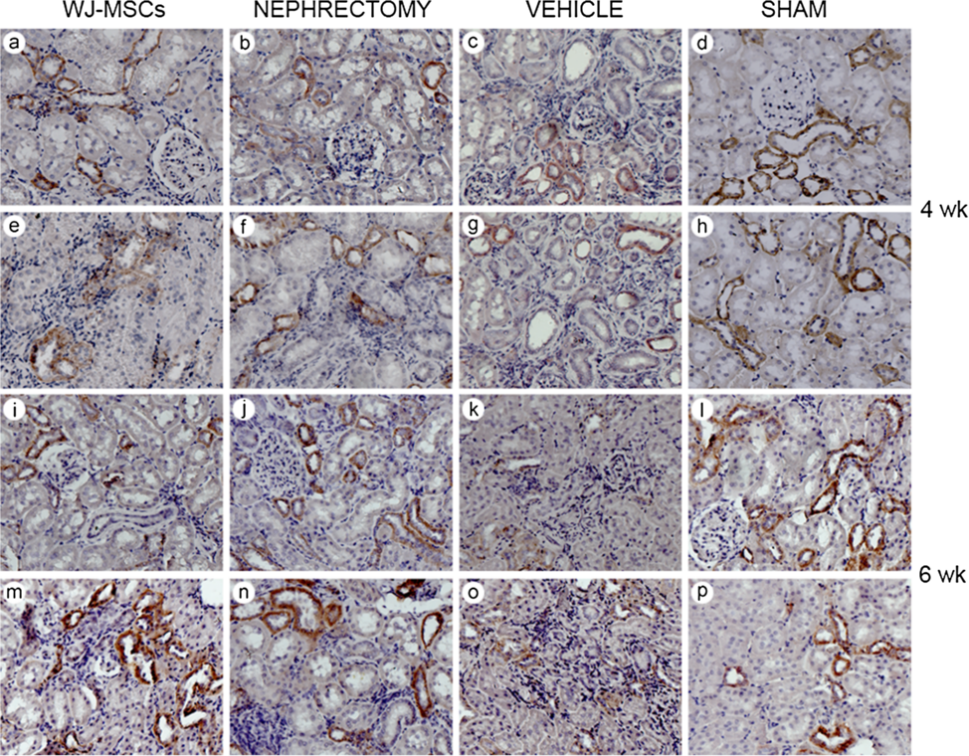
**Representative micrographs illustrating E-cadherin expression in kidney tissues.** Positive expression of E-cadherin was sparse in kidney tissue sections from vehicle-injected IRI rats, especially at 6 weeks, whereas abundant E-cadherin-positive expression was noted in those of sham-operated rats. In the presence of cell treatment or nephrectomy, a lot of loss of E-cadherin induced by IRI did not occur. The original magnification is ×400. **(a)** through **(h)**, photographs showing E-cadherin expression in kidney tissue sections at 4 weeks after different treatment; **(i)** through **(p)**, photographs showing E-cadherin expression in kidney tissue sections at 6 weeks after different treatment.

Tubular EMT is characterized by loss of epithelial proteins, such as E-cadherin, and acquisition of new mesenchymal markers, including α-SMA [17]. Thus, the upregulation of α-SMA/E-cadherin expression was adopted as an indicator of tubular EMT. Induction of IRI caused a substantial time-dependent upregulation of α-SMA/E-cadherin expression in injured kidney, as determined with Western blot (Figure 3), indicative of the development and progression of tubular EMT. However, in the presence of WJ-MSCs or nephrectomy, the upregulation was robustly frustrated. This finding was further corroborated with immunostaining for α-SMA and E-cadherin (Figures 4 and 5). The expression of α-SMA is a key feature of myofibroblasts [18], the key effector cells in the pathogenesis of fibrosis [19]. Therefore, the decline in myofibroblast accumulation undoubtedly accounts for the decrease in collagen production.

At the initial stage of IRI, through native and foreign HGF induction in injured TECs, cell administration substantially shifted the balance between HGF and TGF-β1 in injured kidney in favor of HGF activity, facilitating immediate injury repair and thereby preventing the shift of the balance toward TGF-β1 during fibrogenesis

**Figure 6:**
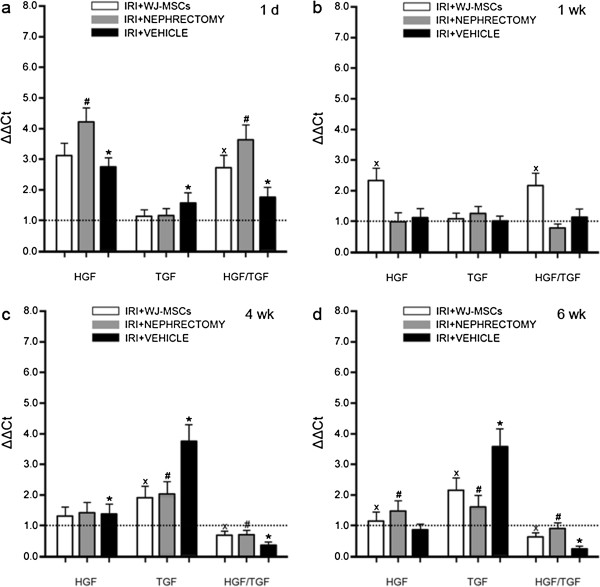
**Hepatocyte growth factor (HGF) or transforming growth factor (TGF)-β1 gene expression in kidney tissues.** At the incipient stage (1 day), IRI triggered the upregulation of HGF/TGF-β1 expression, further promoted by nephrectomy or WJ-MSCs. At 1 week, WJ-MSCs remarkably induced the upregulation of HGF expression in damaged kidney tissue, thereby preventing the expression of HGF/TGF-β1 from decline to baseline. At 4 or 6 weeks, the HGF/TGF-β1 expression was substantially downregulated in vehicle-injected IRI rats, whereas this change was robustly prevented by cell injection or nephrectomy. The Ct (threshold cycle) for the target gene and β-actin was determined for each sample. The quantification of the target gene was normalized by β-actin. HGF/TGF-β1 was generated by referencing HGF expression to TGF-β1 expression. Gene expression in sham-treated samples was regarded as the calibrator (dotted line).The relative expression of HGF, TGF-β1, or HGF/TGF-β1 was calculated by 2^-ΔΔCt^. Data are expressed as the mean of 2^-ΔΔCt^ ± SD of three rats for each experimental condition. x*P* < 0.05, IRI+WJ-MSCs versus IRI+VEHICLE; #*P* < 0.05, IRI+NEPHRECTOMY versus IRI+VEHICLE; ∗*P* < 0.01, IRI+VEHICLE versus SHAM. **(a)** through **(d)** graphs representing relative expression of HGF/β-actin, TGF-β1/β-actin, and HGF/TGF-β1 at 1 day, 1, 4, and 6 weeks after treatment, respectively.

**Figure 7:**
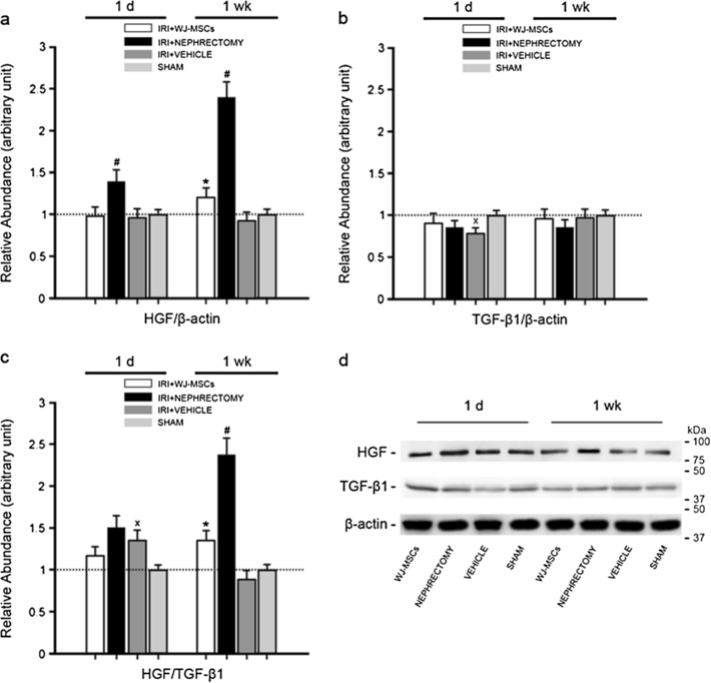
**Densitometric analysis and gel photograph of HGF or TGF-β1 protein expression in kidney tissues at 1 day or 1 week after treatment.** At 1 day, exposure to IRI stimulated the upregulation of HGF/TGF-β1 expression. However, by 1 week, the HGF/TGF-β1 expression came back to its normal status. By contrast, cell treatment or nephrectomy significantly upregulated the HGF/TGF-β1 expression through induction of HGF expression in damaged kidney, in favor of immediate injury repair. The density of each band was determined. Values in the graph are expressed as densitometric ratios of HGF/β-actin, TGF-β1/β-actin, or HGF/TGF-β1 as folds over control (sham-operated samples; dotted line). Data are shown as mean ± SD of three kidney samples for each experimental condition. *P < 0.05, IRI+WJ-MSCs versus IRI+VEHICLE; #*P* < 0.001, IRI+NEPHRECTOMY versus IRI+VEHICLE; x*P* < 0.05, IRI+VEHICLE versus SHAM. **(a)** Relative abundance of HGF/β-actin; **(b)** relative abundance of TGF-β1/β-actin; **(c)** relative abundance of HGF/TGF-β1; **(d)** gel photograph of HGF, TGF-β1, and β-actin protein expression.

**Figure 8:**
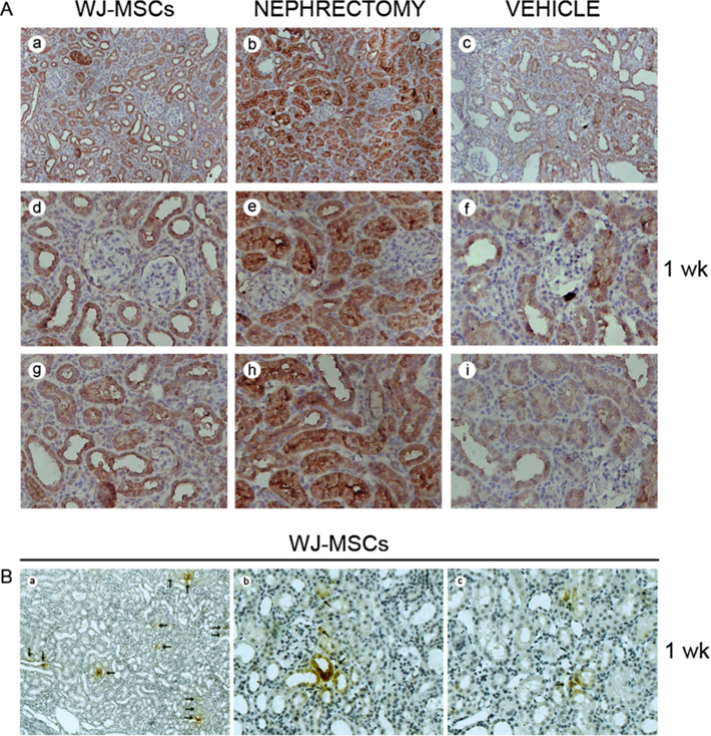
**Hepatocyte growth factor (HGF) expression in injured kidney tissues at 1 week after intervention. (A)** Representative micrographs illustrating HGF expression in injured kidney tissues. The overwhelming majority of positive staining resided in tubular cells. HGF staining in tubular cells was substantially intensified in cases of cell injection or nephrectomy. **(a)** through **(c)** Magnification ×200; **(d)** through **(i)** Magnification ×400. **(B)** Representative micrographs illustrating human HGF expression in injured kidney tissues. No positive-staining TECs were identifiable in kidney sections from sham-operated animals or IRI animals receiving vehicle injection (data not shown). By contrast, at 1 week after injection, positive staining (black arrows) was detected in kidney sections from cell-injected animals, mostly residing in cytoplasm of tubular cells. (**a**) Magnification ×100; **(b)** and **(c)** Magnification ×400.

At a very early stage (1 day), unilateral IRI initiated the upregulation of HGF/TGF-β1 expression in support of injury repair [8] (Figures 6 and 7). Kidney excision or WJ-MSCs further induced the upregulation of HGF/TGF-β gene expression rather than protein expression (Figures 6 and 7). At 1 week, the expression of HGF/TGF-β1 in damaged kidney tissue declined to baseline (Figures 6 and 7). Because of induction of HGF-expression upregulation, this alteration did not occur in cases of cell administration or nephrectomy (Figures 6 and 7). Subsequently, we deliberately separated native HGF expression from foreign HGF. By use of specific primers for detection of rat or human HGF mRNA, we found that rat HGF mRNA was remarkably upregulated by cell treatment, whereas human HGF mRNA was undetectable at this time (1 week) (data not shown). In addition, immunostaining pictures showed that HGF expression was mainly present in rat TECs (Figure 8A). As a response to cell administration, a significant intensification of HGF-staining occurred in TECs (Figure 8A). More intriguingly, human HGF protein expression in rat TECs was unambiguously identified in cell-injected animals, whereas no positive staining for human HGF existed in kidney sections from vehicle-injected or sham-operated animals (Figure 8B). Moreover, at day 1, human HGF mRNA was found in injured kidney tissues, as indicated by real-time PCR. This indicates that WJ-MSCs induce native and foreign HGF expression in injured TECs, thereby leading to the shift of the balance between HGF and TGF-β1 toward the side of HGF, which provides a favorable milieu for repairing injury.

With the progression of chronic kidney injury triggered by IRI, the expression of HGF/TGF-β1 gradually turned to downregulation, facilitating tubular cell phenotype transition, as well as the formation of fibrotic lesion (Figure 6) [8]. By contrast, the administration of WJ-MSCs or excision of intact kidney dramatically prevented the downregulation of HGF/TGF-β1 expression via inhibition of TGF-β1 expression (Figure 6).

### *In vitro*, CM from WJ-MSCs induces the synthesis of native and foreign HGF in hypoxia-injured rat TECs

**Figure 9:**
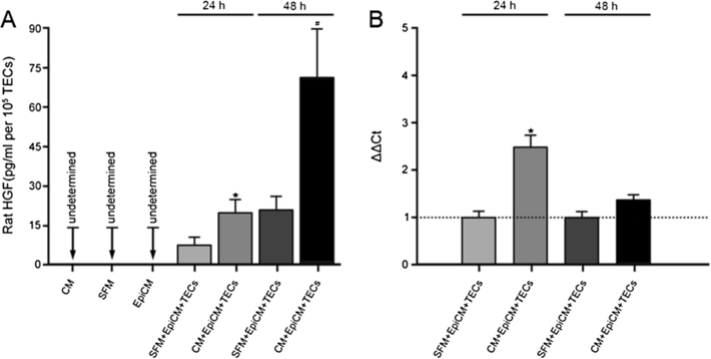
**Rat hepatocyte growth factor (HGF) synthesis in the injured rat TECs after 24 hours or 48 hours of incubation with CM or not. (A)** Release of rat HGF by rat TECs. A total absence of rat HGF appeared in the medium conditioned by WJ-MSCs, SFM, or EpiCM. After 24 hours or 48 hours of incubation, CM strongly amplified the release of rat HGF by rat TECs subjected to hypoxia injury when compared with SFM. The ELISA results were normalized by cell numbers. Data are expressed as mean ± SD of three experiments. ∗*P* = 0.003, CM+EpiCM+TECs versus SFM+EpiCM+TECs; #*P* < 0.05, CM+EpiCM+TECs versus SFM+EpiCM+TECs; CM, conditioned medium from WJ-MSCs; SFM, serum-free medium; EpiCM, epithelial cell medium. **(B)** Rat HGF gene expression in rat TECs. Rat HGF gene expression in hypoxia-injured rat TECs was enhanced by CM from WJ-MSCs at 24 hours or 48 hours of incubation. Statistical significance was not achieved at 48 hours. Rat HGF mRNA was undetermined in WJ-MSCs, as a negative control. The Ct (threshold cycle) for rat HGF and β-actin was determined for each sample. The quantification of HGF was normalized by β-actin. HGF expression in TECs incubated without CM was regarded as the calibrator (dotted line).The relative expression of the target gene was calculated by 2^-ΔΔCt^. Data are expressed as mean of 2^-ΔΔCt^ ± SD of three experiments. ∗*P* < 0.05, CM+EpiCM+TECs versus SFM+EpiCM+TECs.

When rat TECs subjected to hypoxic injury were incubated with CM for 24 hours or 48 hours, a marked increase in rat HGF level was detected with ELISA in the supernatants of TECs (Figure 9A). Moreover, gene expression of rat HGF in TECs was enhanced by CM at either point in time, although a significant difference was not achieved at 48 hours (Figure 9B). These results suggest that CM not only induces native HGF expression in rat TECs but also amplifies its release.

**Figure 10:**
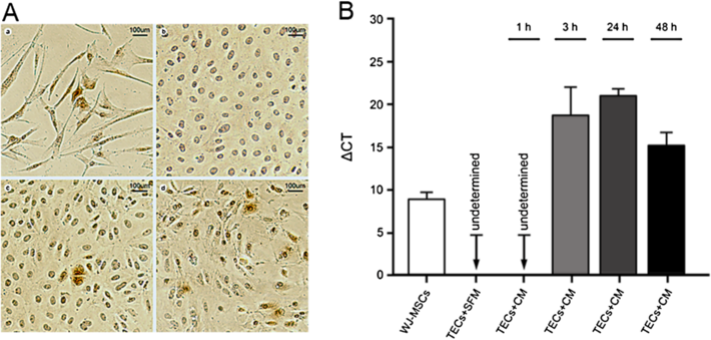
**Human hepatocyte growth factor (HGF) mRNA and protein present in injured rat TECs after exposure to CM. (A)** Representative micrographs indicating human HGF expression in the damaged rat TECs**.** Human HGF was present in cytoplasm of most of the WJ-MSCs and was used as a positive control. Rat TECs without exposure to CM, as a negative control, did not contain this heterogeneous protein. By contrast, it magically appeared in TECs exposed to CM for 24 hours or 48 hours. **(a)** WJ-MSCs; **(b)** TECs without exposure to CM; **(c)** TECs after 24 hours of exposure to CM; (**d**) TECs after 48 hours of exposure to CM; **(B)** Human HGF mRNA entering the injured rat TECs. Human HGF mRNA was detected in rat TECs exposed to CM for 3 hours or longer. Human HGF expressions in WJ-MSCs and rat TECs without exposure to CM were used as positive and negative controls, respectively. The Ct (threshold cycle) for human HGF gene and β-actin gene (rat or human) was determined for each cell sample. The quantification of the target gene was normalized by β-actin. Data are expressed as mean ± SD of three experiments.

Inspired by the *in vivo* evidence, we attempted to determine whether CM from WJ-MSCs induces foreign HGF synthesis in damaged rat TECs. At 24 hours or 48 hours after exposure to CM, human HGF resident in TECs was distinctly identified by immunostaining (Figure 10A). In addition, the presence of human HGF mRNA in TECs was evidenced by real-time PCR as early as 3 hours after exposure (Figure 10B). On the basis of these findings, we are convinced that a foreign HGF gene transcript existing in CM enters rat TECs subjected to hypoxia injury and then is translated into the objective protein.

## Discussion

In some AKI animal models, the impact of MSCs on AKI-induced chronic kidney disease (CKD) has been well documented [20,21]. Consistent with these reports, WJ-MSCs delivered at 2 days after IRI unambiguously protected IRI animals against the development of fibrotic lesions.

In this study, IRI animals undergoing contralateral nephrectomy were regarded as positive controls to facilitate the understanding of potential mechanisms. Some evidence gives support to this choice. First, it is increasingly appreciated that aberrant incomplete repair triggered by AKI contributes to CKD, whereas complete repair leaves no lasting evidence of damage [2,22]. In the wake of unilateral nephrectomy, renal regeneration is initiated [23], thus leading to more complete repair in the injured remaining kidney. It has been reported that renal fibrosis triggered by unilateral IRI can be abolished by contralateral nephrectomy [24]. As expected, nephrectomy undergone at 2 days after ischemia rescued the fibrosis in the ischemic kidney.

In spite of unresolved tubular cell mechanisms implicated in the fibrogenetic process, tubular EMT has been proposed as one of the crucial mechanisms [25-27]. In this study, we succeeded in ascertaining the intimate association of tubular EMT with fibrogenesis. IRI initiated and fueled the process of EMT in parallel with the progression of fibrogenesis, whereas inhibition of EMT by nephrectomy or cell administration coincided with rescue of renal fibrosis.

HGF and TGF-β1 function as the Yin and Yang of tissue fibrotic signals that elicit opposite actions [8]. To a large extent, the reciprocal balance of TGF-β1 and HGF determines the sequelae of tissue injury. The predominance of HGF promotes tissue repair, whereas tissue scarring occurs in the advantage of TGF-β1 over HGF. Mounting evidence establishes the pivotal role for TGF-β/Smad signaling in mediating EMT [28], whereas tubular EMT can be blocked by HGF through inducing gene expression of the Smad co-repressor SnoN [7]. In cultured proximal tubular epithelial cells, HGF completely abolishes TGF-β1-triggered induction of EMT [29].

By contrast, blocking the action of HGF by a neutralizing antibody induces α-SMA expression in renal tubular epithelium [30]. The opposite effects of HGF and TGF-β1 are also reflected by their reciprocal regulation of each other [31-33]. However, Esposito *et al*. [34] found that HGF acts as anti-fibrotic factor reducing TGF effect only on quiescent renal tubular (HK-2) cells. On proliferating cells, HGF increases TGF expression. But this *in vitro* study needs further confirmation. In view of this rational evidence, one strategy devoted to maintenance of the delicate balance between TGF-β1 and HGF may be favorable for the retardation of tubular EMT.

Our observations provided further support for this notion. In this study, after exposure to IRI for 4 or 6 weeks, the balance between HGF and TGF-β1 in the damaged kidney turned toward TGF-β1, accompanying the development of EMT. On the contrary, the recovery of the biased balance, just as in cases of intact kidney removal or cell delivery, was followed by the EMT delay. Therefore, it is reasonable that either WJ-MSCs or nephrectomy hinders tubular EMT through restoration of the disturbed balance of HGF/TGF-β1 during fibrogenesis.

We were interested in the mechanism whereby WJ-MSCs adjusted the HGF/TGF-β1 balance. To facilitate understanding, we first made clear how nephrectomy exerted an effect on this balance. In our study, at 1 day or 1 week after nephrectomy, HGF expression in the ischemic remaining kidney was substantially intensified in agreement with the role of HGF in nephrectomy-stimulated regrowth [23]. Because of the multifaceted beneficial functions of HGF [13], acute injury repair is accelerated, thereby alleviating the ensuing chronic kidney injury, which inevitably leads to recovery of the skewed balance at the fibrotic stage. In our opinion, induction of kidney *in situ* HGF expression at the incipient period of IRI is one of the crucial mechanisms.

Interestingly, WJ-MSCs were involved in the balance modulation via a similar mechanism. At 1 week after injection, cell injection robustly induced gene and protein expression of HGF in the ischemic kidney and thus led to HGF dominance in the balance. By means of immunostaining, we not only identified tubular cells as the main cells expressing HGF but also detected the intensification of HGF-staining in renal tubular cells after cell treatment. Foreign HGF protein expression in injured tubular cells was also distinctly determined. These findings indicate that WJ-MSCs tip the balance through induction of native and foreign HGF synthesis in tubular cells.

More solid evidence is derived from *in vitro* study. After 24 or 48 hours of incubation, the media conditioned by WJ-MSCs not only substantially induced the upregulation of rat HGF mRNA expression in TECs subjected to hypoxia injury but also greatly stimulated the release of rat HGF by rat TECs. Moreover, human HGF protein was detected in rat TECs exposed to CM, which along with the presence of human HGF mRNA in TECs as early as 3 hours after exposure, shows that foreign HGF transcripts existing in CM may enter rat TECs and then be translated into the protein.

As supportive of our findings, in the animal models of ischemia or cisplatin-induced AKI, the administration of human MSCs stimulated the upregulation of HGF mRNA expression in injured murine kidney tissues [14,35]. However, distinct from this study, the previous studies neither deliberately distinguished native HGF from foreign HGF nor determined the target cells where HGF was induced. It is noteworthy that it is the first study documenting foreign HGF expression in injured host tubular cells under induction of heterogeneous MSCs.

**Figure 11:**
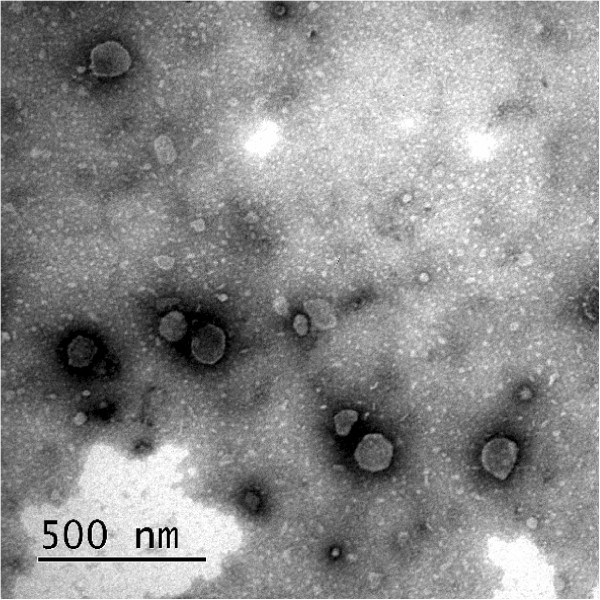
**Representative micrographs of scanning electron microscopy of microvesicles (MVs) isolated from CM from WJ-MSCs.** MVs show a spheroid shape with size ranging from 30 to 500 nm.

Growing evidence suggests that microvesicles (MVs) released by MSCs can deliver mRNA, regulatory micro-RNA and transcriptional factor to injured tissue cells, thus leading to alteration of cell function [10,11]. We think that MVs secreted by WJ-MSCs in an endocrine manner may be implicated in the induction of native and foreign HGF synthesis in renal tubular cells (target cells). It is feasible for this event to occur. In an *in vitro* study, incubation of murine TECs with different doses of MVs derived from human bone marrow MSCs induced *de novo* expression of some human proteins in murine tubular cells [36]. The ascertainment of this mechanism will be the goal of the future research. We have isolated MVs from CM from WJ-MSCs and have partly determined its characterization (Figure 11), but we still have a long way to go.

## Conclusions

Against the background of ischemic AKI, WJ-MSCs contribute to restoration of the biased balance of HGF/TGF-β1 during fibrogenesis via induction of native and foreign HGF synthesis in injured tubular cells at the initial stage of AKI, which consequently results in the EMT delay and alleviation of renal fibrosis.

## Abbreviations

AKI: Acute kidney injury; CM: Conditioned medium; EMT: Epithelial-mesenchymal transition; EpiCM: Epithelial cell medium; HGF: Hepatocyte growth factor; IRI: Ischemia-reperfusion injury; MSC: Mesenchymal stromal cell; MV: Microvesicles; NuMA: Nuclear mitotic apparatus protein; RT-PCR: Real-time polymerase chain reaction; SD: Sprague–Dawley; SFM: Serum-free medium; α-SMA: α-smooth muscle actin; TEC: Tubular epithelial cell; TGF-β1: Transforming growth factor-beta1; WJ-MSC: Wharton jelly-derived mesenchymal stromal cell

## Competing interests

The authors declare that they have no competing interests.

## Authors’ contributions

TD conceived and designed the experiments, performed the experiments, analyzed the data, and wrote the paper. XYZ and JC conceived of, designed, and performed the experiments, and analyzed the data. JC and TD edited the figures. SW, LZ, and GQJ performed the experiments. JZ and GHL conceived of and designed the experiments. YJZ conceived of and designed the experiments, analyzed the data, and revised the paper. SJX revised the paper. All authors read and approved the final manuscript.
